# Supplementary material for: Interdigitating Modules for Visual Processing During Locomotion and Rest in Mouse V1
Source: bioRxiv. 2025 Feb 25:2025.02.21.639505. Preprint. [Version 1] doi: 10.1101/2025.02.21.639505 (PMC11888233; doi:10.1101/2025.02.21.639505)
Supplement: Supplement 1 [file NIHPP2025.02.21.639505v1-supplement-1.pdf]

bioRxiv preprint doi: https://doi.org/10.1101/2025.02.21.639505; this version posted February 25, 2025. The copyright holder for this preprint (which was not certified by peer review) is the author/funder, who has granted bioRxiv a license to display the preprint in perpetuity. It is made available under aCC-BY 4.0 International license.

Supplemental Figures

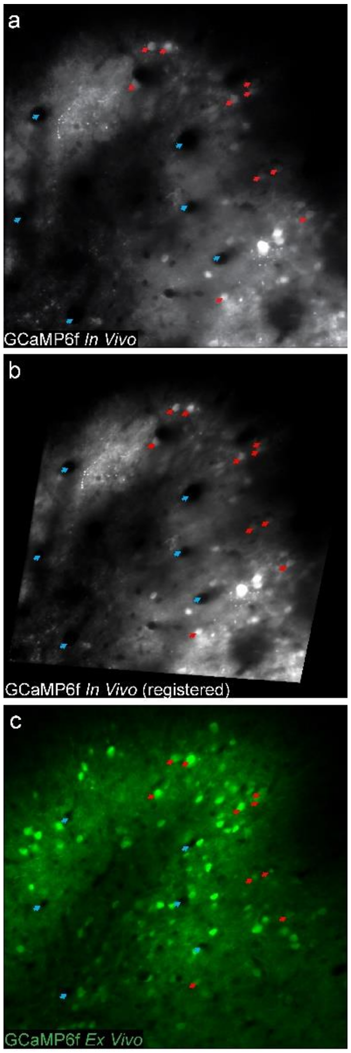

Figure 1 Supplement 1 Demonstration of image registration of *in vivo* recording planes with *ex vivo* sections. (**a**) Non-registered image of time-averaged GCaMP6f fluorescence across a session in the recording plane. Cyan arrows point to blood vessels and red arrows point to GCaMP6f-labeled cells which were clearly identifiable in both this *in vivo* image and the corresponding *ex vivo* section (panel **c**). These points were manually chosen in both images and used as fiducial points for registration. (**b**) Registered version of the *in vivo* image. Registration consisted almost entirely of rotation, with very little distortion or change in relative distances between points in the *in vivo* image. The Matlab function 'fitgeotrans' was used by inputting point pairs, including those labeled with red arrows, from the non-registered *in vivo* image (panel **a**) and the *ex vivo* image (panel **c**). *In vivo* landmarks were used as "moving points" and *ex vivo* landmarks were used as "fixed points." (**c**) GCaMP6f fluorescence in *ex vivo* section at same location as *in vivo* recording. Cyan and red arrows are in the same locations and point to the same anatomical structures in this section and in the registered *in vivo* image (panel **b**), demonstrating that the registration process was successful in precisely aligning locations across the two conditions.

bioRxiv preprint doi: https://doi.org/10.1101/2025.02.21.639505; this version posted February 25, 2025. The copyright holder for this preprint (which was not certified by peer review) is the author/funder, who has granted bioRxiv a license to display the preprint in perpetuity. It is made available under aCC-BY 4.0 International license.

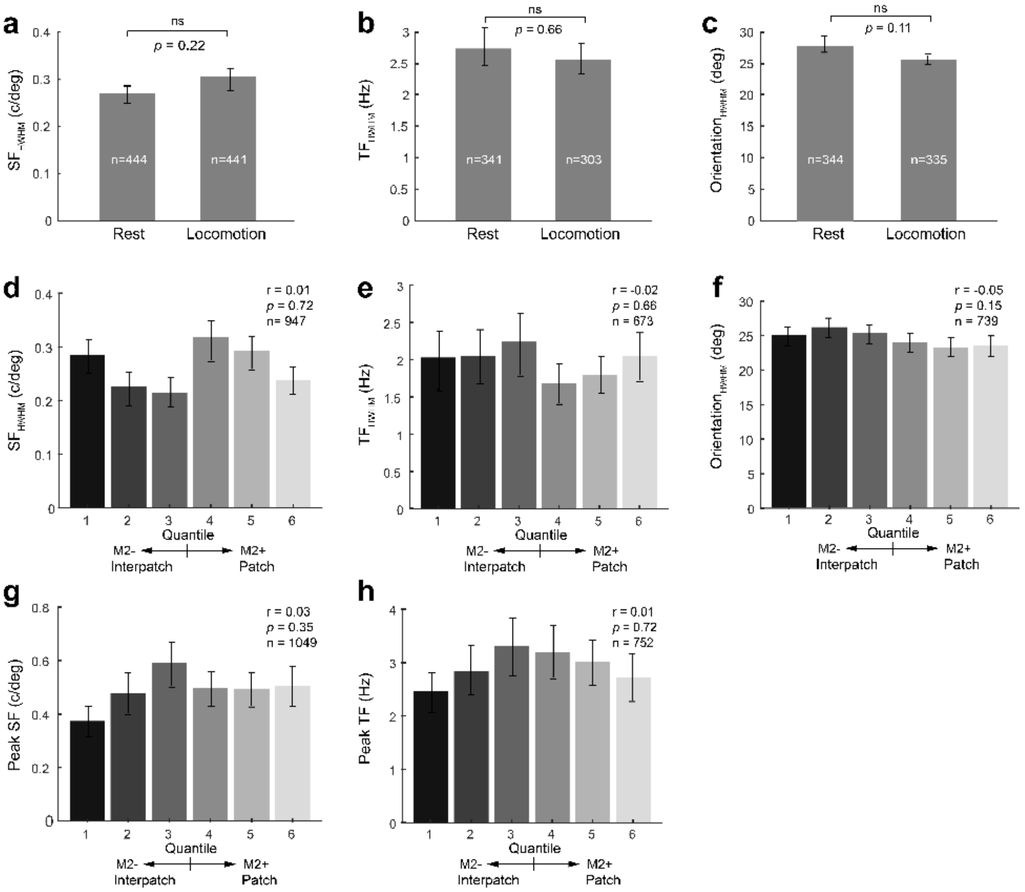

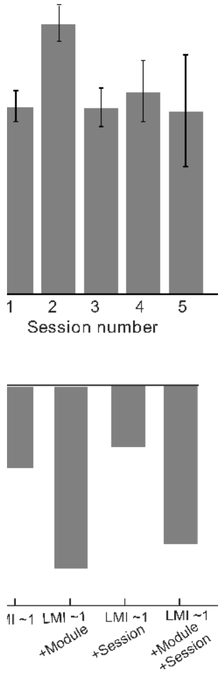

ure 2 Supplement 2.1

Figure 2

Supplement 2.1 Effect of recording session number on locomotion modulation. (**a**) Mean LMI across cells in all mice according to recording session number. Cells were divided into 5 groups depending on how many experimental sessions the mouse had experienced prior to that cell being recorded. Experience in prior recording sessions neither systematically increased nor decreased LMI of cells (r = 0.006, *p* = 0.882, Pearson correlation). (**b**) Relative goodness of fit of linear models for LMI which either included or excluded session number as an independent variable. Akaike Information Criterion (AIC) was used to compare model suitability. Using session number as the only predictor (3rd model) was inferior to a model using only a fixed intercept (1st model). Similarly, adding session number as a predictor (4th model) to a model using M2 module as a predictor (2nd model) reduced the model suitability.

Figure 2 Supplement 2.2 Effects of locomotion and M2 module on visual tuning parameters of neural responses. (**a-c**) Half-width at half maximum (HWHM) of spatial frequency (SF), temporal frequency (TF), or orientation tuning computed from locomotion (mean forward velocity > 0.1cm/s) and stationary trials (mean forward velocity < 0.1cm/s). For each cell, a

bioRxiv preprint doi: https://doi.org/10.1101/2025.02.21.639505; this version posted February 25, 2025. The copyright holder for this preprint (which was not certified by peer review) is the author/funder, who has granted bioRxiv a license to display the preprint in perpetuity. It is made available under aCC-BY 4.0 International license.

tuning curve while stationary or during running were computed. Locomotion state did not change the HWHM of any stimulus parameter ($p > 0.05$, paired t-test). (**d-f**) HWHM of SF, TF and orientation tuning of cells in M2+ patches and M2- interpatches. M2+ patch and M2- interpatch cells did not show differences in HWHM for any tuning parameter ($p > 0.05$, t-test). (**g-h**) Peak SF and TF of cells in M2+ patches and M2- interpatches. M2+ patch and M2- interpatch cells did not show differences in peak SF or TF ($p > 0.05$, Pearson correlation).

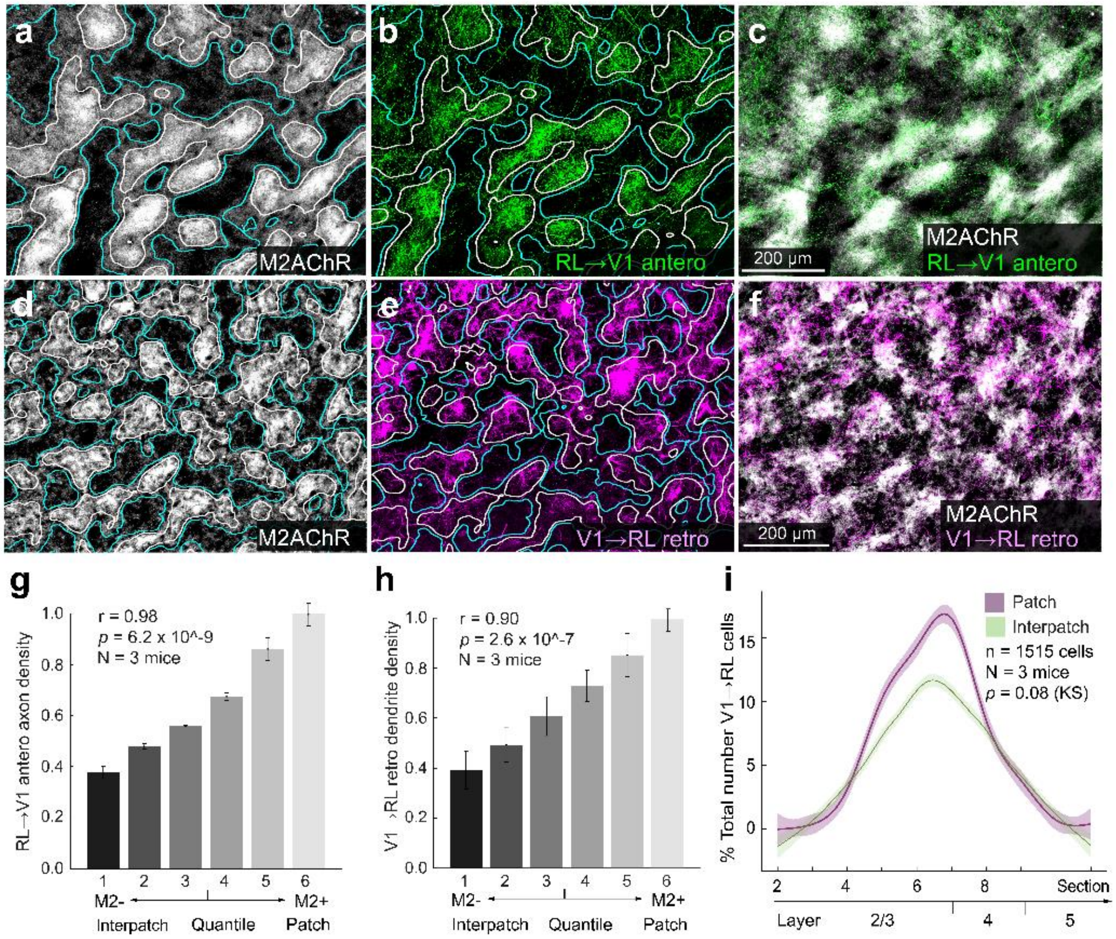

Figure 5 - Supplement 1

Figure 5 Supplement 1 Modular organization of connections between V1 and RL. (**a**) Tangential section through L1 of V1 of Ai9 mouse stained with antibody against M2. M2+ patches (white) are outlined by white contours, M2- interpatches by cyan lines. (**b**). Anterogradely AAV-labeled RL→V1 axons (green) in L1 of V1 shows preferential termination in M2+ patches. (**c**) Overlay of panel a and b. (**d-f**) Apical dendrites of retrogradely AAV-labeled V1→RL-projecting neurons in L1 preferentially branch in M2+ patches. (**g**) Labeling

bioRxiv preprint doi: https://doi.org/10.1101/2025.02.21.639505; this version posted February 25, 2025. The copyright holder for this preprint (which was not certified by peer review) is the author/funder, who has granted bioRxiv a license to display the preprint in perpetuity. It is made available under aCC-BY 4.0 International license.

density of axons in different M2 quantiles. Pearson correlation (r), error bars ±SEM. (**h**) Labeling density of dendrites in different M2 quantiles. Pearson correlation (r), error bars ±SEM. (**i**) Percent of retrogradely AAV-labeled V1→RL-projection neurons in M2+ patches (magenta) and M2- interpatches (green) in different layers of V1. Shading ±SEM. KS test.

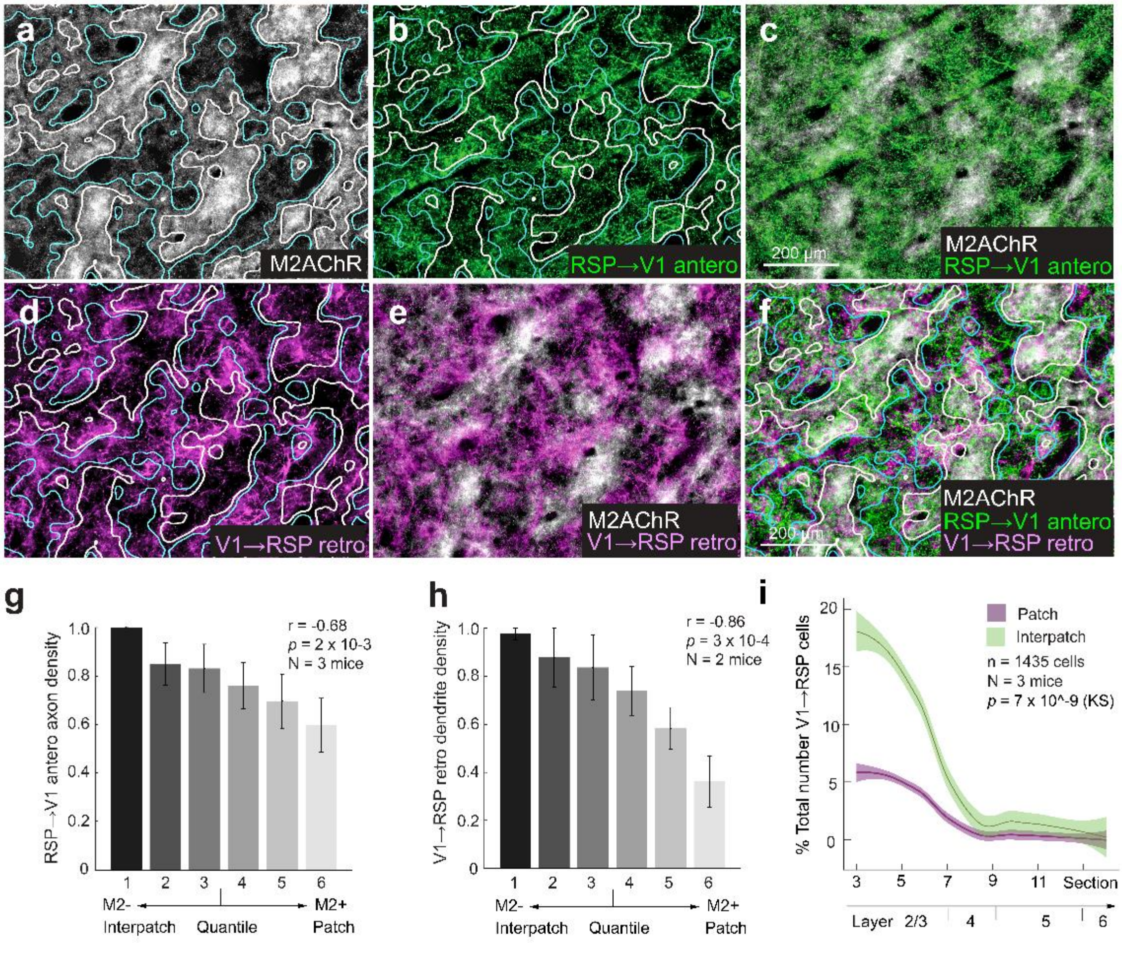

Figure 6 Supplement 1

Figure 6 Supplement 1 Modular organization of connections between V1 and RSP. (**a**) Tangential section through L1 of V1 of Ai9 mouse stained with antibody against M2. M2+ patches (white) are outlined by white contours, M2- interpatches by cyan lines. (**b**, **c**). Anterogradely AAV-labeled RSP→V1 axons (green) in L1 of V1 shows preferential termination in M2- interpatches. (**d-f)** Apical dendrites of retrogradely AAV-labeled V1→RSP-projecting neurons in L1 preferentially branch in M2- interpatches. (**g**) Labeling density of axons in different M2 quantiles. Pearson correlation (r), error bars ±SEM. (**h**) Labeling density of

bioRxiv preprint doi: https://doi.org/10.1101/2025.02.21.639505; this version posted February 25, 2025. The copyright holder for this preprint (which was not certified by peer review) is the author/funder, who has granted bioRxiv a license to display the preprint in perpetuity. It is made available under aCC-BY 4.0 International license.

dendrites in different M2 quantiles. Pearson correlation (r), error bars ±SEM. (**i**) Percent of retrogradely AAV-labeled V1→RSP-projection neurons in M2+ patches (magenta) and M2- interpatches (green) in different layers of V1. Shading ±SEM. KS test.

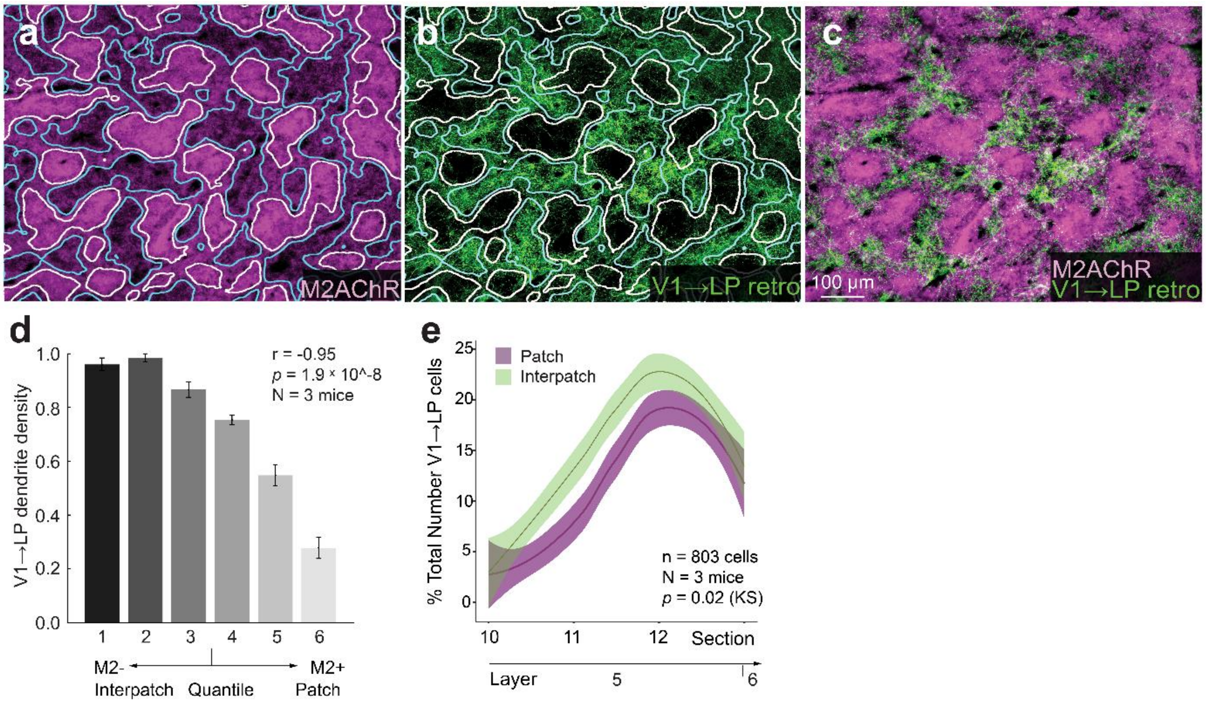

Figure 6 Supplement 2

Figure 6 Supplement 2 Modular organization of connections between LP and V1. (**a**) Tangential section through L1 of V1 of Ai9 mouse stained with antibody against M2. M2+ patches (magenta) are outlined by white contours, M2- interpatches by cyan lines. (**b**, **c)** Apical dendrites (green) of retrogradely AAV-labeled V1→LP-projecting L5 neurons preferentially branch in M2- interpatches of L1. (**d**) Labeling density of dendrites in different M2 quantiles shows that dendritic branches are denser in M2- interpatches. Pearson correlation (r), error bars ±SEM. (**e**) Retrogradely labeled V1→LP-projecting cell bodies show that cell bodies in L5 are preferentially (p = 0.02, KS) aligned with M2- interpatches (green). Shading ±SEM. KS test.
